# Information-Accessing Behavior during Zika Virus Outbreak, United States, 2016

**DOI:** 10.3201/eid2609.191519

**Published:** 2020-09

**Authors:** Rachael Piltch-Loeb, David Abramson

**Affiliations:** New York University, New York, New York, USA

**Keywords:** Zika virus, risk communication, vector-borne infections, viruses, information, media, news, United States

## Abstract

We used latent class analysis to examine Zika virus–related information-accessing behavior of US residents during the 2016 international outbreak. We characterized 3 classes of information-accessing behavior patterns: universalists, media seekers, and passive recipients. Understanding these patterns is crucial to planning risk communication during an emerging health threat.

During the past 15 years, new media platforms have emerged as routine channels of health communication. Little is known about how persons navigate this dynamic and complex information landscape, especially during an emerging health threat with little scientific certainty and few or no medical countermeasures (*1*,*2*). The 2016 Zika virus outbreak provides for an examination of how people interact with this dynamic information landscape. As scientific understanding of the virus evolved, so did Zika risk communication strategies. Previous reports have identified public sources of Zika information but have not considered the public’s information-accessing behavior (*3*,*4*). We used latent class analysis (LCA) to characterize and differentiate types of information-accessing behavior and identify how these behavioral patterns shifted during the 2016 Zika virus outbreak.

LCA identifies clusters within the population on the basis of participants’ responses to observed variables (*5*,*6*). We collected and pooled data from 3 representative samples of US households drawn from fully replicated, single-stage, random-digit dialing samples of households supplemented by lists of randomly generated cell phone numbers. The survey had a 4%–6% response rate. We conducted the surveys in April–May (1,233 participants), July–August (1,231 participants), and October–November (1,234 participants) of 2016.

The survey analyzed access to 6 categories of information sources: news (online or print); television or radio; social media, such as Facebook, YouTube, Reddit, or other apps; personal physician; government agencies; and friends, family, or co-workers. We used these data to form 6 binary variables indicating access to each category of information source. We then used these variables to determine 3 classes of information-accessing behavior.

In accordance with the best practices suggested by Nylund et al. (*7*), we used 6 criteria to determine the optimal number of classes (Appendix). New York University’s Institutional Review Board approved this research.

Our LCA results suggested that information-accessing behaviors could be grouped into 3 distinct classes: universalists, media seekers, and passive recipients. We sorted each participant into a class on the basis of the number of sources he or she had accessed (Figure). Class 1 comprised universalists, that is, participants who actively accessed information from all sources included in the survey. Class 2 comprised media seekers, that is, participants who primarily accessed information from mass media. Class 3 comprised passive recipients of information; these participants accessed the fewest number of sources and had the highest probability of seeking information from broadcast media. Class membership was not necessarily static; an individual participant might exhibit different information-accessing behaviors at different time points within the Zika outbreak.

**Figure:**
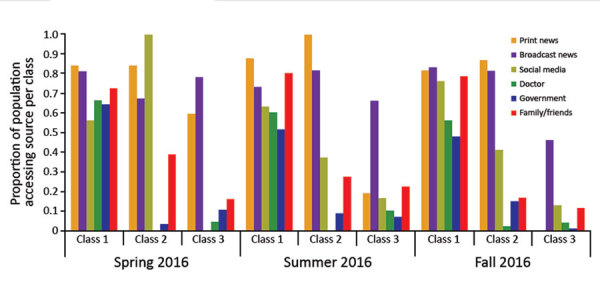
Latent classes of information access for Zika virus, United States, 2016. Proportion of the population that used a given category of information source in each class, across 3 time points of data collection.

The acquisition patterns of Zika information shifted across time. At the first time point (April–May 2016), universalists constituted 23.0% of the US population, media seekers 20.7%, and passive recipients 54.3%. At the second time point (July–August 2016), universalists constituted 13.8% of the population, media seekers 51.5%; and passive recipients 34.8%. At the third time point (October–November 2016), universalists constituted 16.0% of the population, media seekers 52.0%, and passive recipients 32.0%.

As understanding of Zika virus evolved and media coverage shifted, the proportion of the population in each of the identified classes also shifted. Our finding that the proportion of the population in the universalist group was largest at the first time point suggests that in the earlier phases of the Zika outbreak, acquisition of information increased among the most highly attuned portions of the population. We hypothesize that as the mosquito season began, behavior patterns shifted from passive information acquisition to active information acquisition in the shift to media seeking (*8*). This hypothesis explains the shift from the large proportion of passive recipients at the first time point to the larger proportions of media seekers at the second and third time points.

These population shifts suggest large portions of the population were initially passive, perhaps uninterested, recipients of information about Zika. During the course of the surveys, a proportion of passive recipients and universalists may have become media seekers. In addition, we found that early adopters of emerging information could be retransmitters within their networks. Only universalists consistently accessed information from their own social network (including personal contacts and social media), the medical community, and government sources. Further exploration is needed to determine whether these findings are influenced by the actual lack of risk for Zika in the United States or whether they are reflective of larger behavioral patterns. 

Our analysis is limited by the number of information source categories included in the survey and the lack of source specificity. However, our study took a unique approach in characterizing patterns of information-accessing behavior. These findings can be used to inform risk communication strategies designed for population segments with different information-accessing behavior patterns.

Appendix Methods and results of latent class analysis of Zika information-accessing behaviors, United States, 2016
