# Supplementary material for: Information-Accessing Behavior during Zika Virus Outbreak, United States, 2016
Source: Emerg Infect Dis. 2020 Sep;26(9):2290–2. doi: 10.3201/eid2609.191519 (PMC7454085; doi:10.3201/eid2609.191519)
Supplement: Appendix — Methods and results of latent class analysis of Zika information-accessing behaviors, United States, 2016 [file 19-1519-Techapp-s1.pdf]

Article DOI: https://doi.org/10.3201/eid2607.191519

Information-Accessing Behavior During Zika Virus Outbreak, United States, 2016

Appendix

Methods

Data Source

Three cross-sectional samples of the United States population were collected at three distinct time points—Spring (April/May), Summer (July/August), and Fall (October/November) of 2016—which included measures related to source of Zika information. Data was collected from a representative sample of U.S. households collected using a fully-replicated, single-stage, random-digit-dialing (RDD) sample households supplemented by a list of randomly generated cell phone numbers. The first structured telephone survey was of 1,233 U.S. residents, subsequent surveys sampled 1,231 residents and 1,234 residents respectively. Data will be analyzed using complex survey weights so results are representative of the population. Further information on weighting procedures have been described in detail in a previous manuscript (*1*).

Analytic Plan

Statistical methods such as LCA are especially useful to understand if there are underlying subtypes of individuals in the population for the phenomena at hand. LCA will be used to identify if there are "types" of information users within the population. LCA is a statistical tool to study a heterogeneous population consisting of several unidentified groups who behave differently regarding the problem at hand. LCA functions in terms of probability, specifically the probability that an individual belongs to a particular scoring pattern among the observed variables (*2*). While there are multiple interpretations of latent classes, the one being explored here is to classify respondents into being a member of a latent, unobserved class on the basis of their responses to one or more observed variables (*3*). LCA can reduce analytic complexity by identifying patterns of activity (*4*). Cluster analysis and LCA techniques have

been used within the fields of psychology, organizational behavior, and political science, and applied to examining health behaviors (*2,5–11*).

Model Selection

There is some debate in best practices for LCA model selection, especially when applying weighted population estimates when likelihood ratio tests may not be appropriately run since maximum likelihood estimates are not possible (*12*). In accordance with the best practices set out by Nylund, Asparaouhov, & Muthen (2007), several criteria were used to determine the optimal number of classes (*13*). The criteria applied here were:

1. Akaike and Bayesian information criteria (AIC and BIC) (*14*);
2. Lo-Mendell-Rubin adjusted likelihood ratio test;
3. Entropy;
4. the relative size of classes in each model;
5. substantive interpretability;
6. and replication of the LCA solution in all three samples.

Results

LCA results suggested a replicable three-class solution of information users in the population, with classes distinguished by the number of sources accessed. Appendix Tables 1–3 demonstrate the selection criteria used to compare 2–6 classes and reflect the three classes solution had the best goodness of fit at each time point. Results as to the proportion of the population in each class and accessing each source by time point are below in Appendix Tables 4–6.

Sample 1: Spring 2016

The proportion of the population in each of the three-classes is shown in Appendix Table 1. The average latent class probability, an indicator of membership within a latent class, measures how certain an individual is to be in one class compared to another, was high- 0.944, 0.893, and 0.906 respectively. Within Class 1, the probability of getting information from print news was 0.845, broadcast news was 0.814, social media was 0.564, doctor was 0.667,

government was 0.645, and family/friends was 0.729. Within Class 2, the probability of getting information from print news was 0.844, broadcast news was 0.675, social media was 1.00, doctor was 0.00, government was 0.035, and family/friends was 0.390. Within Class 3, the probability of getting information from print news was 0.597, broadcast news was 0.786, social media was 0.004, doctor was 0.047, government was 0.108, and family/friends was 0.164.

Sample 2: Summer 2016

The proportion of the population in each of the three-classes is shown in Appendix Table 2. From time 1 to time 2, the proportion of the population in each class shifted. Class 1, people who sought information from many sources, was 13.8% of the population, Class 2, those who primarily sought information from mass media and social media were 51.5% of the population, and Class 3, the least active information seekers, was 34.7% of the population. The average latent class probability, an indicator of membership within a latent class, measures how certain an individual is to be in one class compared to another, was still high- 0.890, 0.792, and 0.947 respectively. For Class 1, the probability of getting information from print news was 0.881, broadcast news was 0.735, social media was 0.635, doctor was 0.605, government was 0.518, and family/friends was 0.806. Within Class 2, the probability of getting information from print news was 1.00, broadcast news was 0.818, social media was 0.376, doctor was 0.00, government was 0.090, and family/friends was 0.277. Within Class 3, the probability of getting information from print news was 0.193, broadcast news was 0.665, social media was 0.169, doctor was 0.105, government was 0.073, and family/friends was 0.227.

Sample 3: Fall 2016

The proportion of the population in each of the three-classes is shown in Appendix Table 3. The proportion of the population in each class was similar to Sample 2. The average latent class probability, an indicator of membership within a latent class, measures how certain an individual is to be in one class compared to another, was also high- 0.852, 0.860, 0.872- respectively. For Class 1, the probability of getting information from print news was 0.818, broadcast news was 0.834, social media was 0.764, doctor was 0.564, government was 0.482, and family/friends was 0.789. Within Class 2, the probability of getting information from print news was 0.871, broadcast news was 0.816, social media was 0.414, doctor was 0.024, government was 0.151, and family/friends was 0.171. Within Class 3, the probability of getting

information from print news was 0.000, broadcast news was 0.463, social media was 0.131, doctor was 0.044, government was 0.014, and family/friends was 0.118.

References

1. Piltch-Loeb R, Abramson DM, Merdjanoff AA. Risk salience of a novel virus: US population risk perception, knowledge, and receptivity to public health interventions regarding the Zika virus prior to local transmission. PLoS One. 2017;12:e0188666. PubMed https://doi.org/10.1371/journal.pone.0188666

2. Hagenaars JA, McCutcheon AL. Applied latent class analysis. Cambridge: Cambridge University Press; 2002.

3. Goodman LA. The analysis of systems of qualitative variables when some of the variables are unobservable. Part IA modified latent structure approach. American Journal of Sociology. 1974;79:1179–259. https://doi.org/10.1086/225676

4. Lanza ST, Rhoades BL. Latent class analysis: an alternative perspective on subgroup analysis in prevention and treatment. Prev Sci. 2013;14:157–68. PubMed https://doi.org/10.1007/s11121-011-0201-1

5. Bucholz KK, Heath AC, Reich T, Hesselbrock VM, Krarner JR, Nurnberger JI Jr, et al. Can we subtype alcoholism? A latent class analysis of data from relatives of alcoholics in a multicenter family study of alcoholism. Alcohol Clin Exp Res. 1996;20:1462–71. PubMed https://doi.org/10.1111/j.1530-0277.1996.tb01150.x

6. Hudziak JJ, Heath AC, Madden PF, Reich W, Bucholz KK, Slutske W, et al. Latent class and factor analysis of DSM-IV ADHD: a twin study of female adolescents. J Am Acad Child Adolesc Psychiatry. 1998;37:848–57. PubMed https://doi.org/10.1097/00004583-199808000-00015

7. Clatworthy J, Buick D, Hankins M, Weinman J, Horne R. The use and reporting of cluster analysis in health psychology: a review. Br J Health Psychol. 2005;10:329–58. PubMed https://doi.org/10.1348/135910705X25697

8. Kendler KS, Karkowski LM, Walsh D. The structure of psychosis: latent class analysis of probands from the Roscommon Family Study. Arch Gen Psychiatry. 1998;55:492–9. PubMed https://doi.org/10.1001/archpsyc.55.6.492

9. Kiel GC, Layton RA. Dimensions of consumer information seeking behavior. Journal of Marketing Research. 1981;18:233–9. https://doi.org/10.1177/002224378101800210

10. Laska MN, Pasch KE, Lust K, Story M, Ehlinger E. Latent class analysis of lifestyle characteristics and health risk behaviors among college youth. Prev Sci. 2009;10:376–86. PubMed https://doi.org/10.1007/s11121-009-0140-2

11. Punj G, Stewart DW. Cluster analysis in marketing research: review and suggestions for application. Journal of Marketing Research. 1983;20:134–48. https://doi.org/10.1177/002224378302000204

12. Korn EL, Graubard BI. Simultaneous testing of regression coefficients with complex survey data: use of Bonferroni t statistics. Am Stat. 1990;44:270–6. https://doi.org/10.2307/2684345

13. Nylund KL, Asparouhov T, Muthén BO. Deciding on the number of classes in latent class analysis and growth mixture modeling: a Monte Carlo simulation study. Struct Equ Modeling. 2007;14:535–69. https://doi.org/10.1080/10705510701575396

14. Muthén BO. Latent variable mixture modeling. In: Muthén BO, Marcoulides GA, editors. New developments and techniques in structural equation modeling. New York: Psychology Press; 2001. p. 21-54.

Appendix Table 1. Model fit statistics of latent class analysis models on Zika information accessing behaviors. United States, April–May 2016

No. classes	H0 value	H0 scaling correction factor for MLR	AIC	BIC	SBIC	Entropy	Pearson's χ^2 p value	LLR χ^2 p value	Average LC probability for most likely LC	Lo Mendell Rubin p value	Vuong Lo Mendell Rubin p value
2 class model	–4,070.212	2.0027	8,166.425	8,232.948	8,191.655	0.611	<0.01	<0.01	0.844, 0.911	<0.01	<0.01
3 class model	-4,021.901	1.6961	8,083.802	8,186.146	8,122.618	0.804	<0.01	<0.01	0.944, 0.893, 0.906	<0.01	<0.01
4 class model	–3,986.520	1.8278	8,027.040	8,165.204	8,079.441	0.829	0.04	<0.01	0.865, 0.849, 0.906, 0.946	0.36	0.36
5 class model	–3,964.701	1.5854	7,997.402	8,171.387	8,063.388	0.700	0.99	0.03	0.887, 0.816, 0.891, 0.560, 0.889	0.19	0.19

LC, latent class; AIC, Aikake Information criteria; BIC, Bayesian information criteria; MLR, multi-linear regression; LLR, log linear ratio

Appendix Table 2. Model fit statistics of latent class analysis models on Zika information accessing behaviors. United States, July–August 2016

No. classes	H0 Value	H0 Scaling Correction Factor for MLR	AIC	BIC	SBIC	Entropy	Pearson's χ^2 p value	LLR χ^2 p value	Average LC probability for most likely LC	Lo Mendell Rubin p value	Vuong Lo Mendell Rubin p value
2 class model	–3,968.288	2.0518	7,962.576	8,029.068	7,987.775	0.712	<0.01	<0.01	0.758, 0.953	0.02	0.02
3 class model	–3,932.758	1.7150	7,905.516	8,007.812	7,944.283	0.653	<0.01	<0.01	0.890, 0.792, 0.947	0.06	0.06
4 class model	–3,907.997	1.5895	7,869.994	8,008.093	7,922.330	0.738	<0.01	<0.01	0.920, 0.822, 0.864, 0.850	0.13	0.13
5 class model	–3,893.733	1.8898	7,855.467	8,029.369	7,921.371	0.695	<0.01	0.02	0.794, 0.779, 0.712, 0.922, 0.807	0.74	0.74

LC, latent class; AIC, Aikake Information criteria; BIC, Bayesian information criteria; MLR, multi-linear regression; LLR, log linear ratio

Appendix Table 3. Model fit statistics of latent class analysis models on Zika information accessing behaviors. United States, October–November 2016

No. classes	H0 Value	H0 Scaling Correction Factor for MLR	AIC	BIC	SBIC	Entropy	Pearson's χ^2 p value	LLR χ^2 p value	Average LC probability for most likely LC	Lo Mendell Rubin p value	Vuong Lo Mendell Rubin p value
2 class model	–3,934.742	1.7497	7,895.485	7,962.008	7,920.715	0.571	<0.01	<0.01	0.917, 0.797	<0.01	<0.01
3 class model	–3,864.304	1.5507	7,768.608	7,870.952	7,807.423	0.699	0.40	0.03	0.852, 0.860, 0.872	<0.01	<0.01
4 class model	–3,850.260	1.4955	7,754.520	7,892.685	7,806.921	0.645	0.98	0.21	0.818, 0.973, 0.682, 0.846	0.26	0.25
5 class model	–3,839.352	1.3971	7,746.703	7,920.688	7,812.690	0.608	1.00	0.58	0.780, 0.686, 0.812, 0.721, 0.929	0.21	0.22

LC, latent class; AIC, Aikake Information criteria; BIC, Bayesian information criteria; MLR, multi-linear regression; LLR, log linear ratio

Appendix Table 4. Latent class analysis proportions and Zika information sources. United States, April–May 2016

Class category	Class 1	Class 2	Class 3
Proportion of population in class*	0.23030	0.20710	0.54259
Information source, % use (SE)			
Print news	0.845 (0.041)	0.844 (0.072)	0.597 (0.026)
Broadcast news	0.814 (0.040)	0.675 (0.043)	0.786 (0.023)
Social media	0.564 (0.057)	1.000 (0.000)	0.004 (0.095)
Doctor	0.667 (0.071)	0.000 (0.000)	0.047 (0.020)
Government	0.645 (0.050)	0.035 (0.040)	0.108 (0.025)
Family and friends	0.729 (0.060)	0.390 (0.077)	0.164 (0.023)

*<2% of the population could not be adequately sorted into a class

Appendix Table 5. Latent class analysis proportions and Zika information sources. United States, July–August 2016

Class category	Class 1	Class 2	Class 3
Proportion of population in class	0.13775	0.51486	0.34739
Information source, % use (SE)			
Print news	0.881 (0.084)	1.00 (0.000)	0.193 (0.144)
Broadcast news	0.735 (0.060)	0.818 (0.030)	0.665 (0.032)
Social media	0.635 (0.0.88)	0.376 (0.050)	0.169 (0.031)
Doctor	0.605 (0.110)	0.000 (0.000)	0.105 (0.037)
Government	0.518 (0.083)	0.090 (0.024)	0.073 (0.032)
Family and friends	0.806 (0.099)	0.277 (0.047)	0.227 (0.033)

Appendix Table 6. Latent class analysis proportions and Zika information sources. United States, October–November 2016

Class category	Class 1	Class 2	Class 3
Proportion of population in class	0.16000	0.52000	0.32000
Information source, % use (SE)			
Print news	0.818 (0.046)	0.871 (0.086)	0.000 (0.000)
Broadcast news	0.834 (0.039)	0.816 (0.023)	0.463 (0.061)
Social media	0.764 (0.043)	0.414 (0.041)	0.131 (0.042)
Doctor	0.564 (0.116)	0.024 (0.017)	0.044 (0.014)
Government	0.482 (0.074)	0.151 (0.022)	0.014 (0.021)
Family and friends	0.789 (0.065)	0.171 (0.054)	0.118 (0.025)
